# Orientational Effects and Molecular-Scale Thermoelectricity Control

**DOI:** 10.1021/acsomega.4c02141

**Published:** 2024-06-26

**Authors:** Turki Alotaibi, Maryam Alshahrani, Majed Alshammari, Moteb Alotaibi, Taha Abdel Mohaymen Taha, Alaa A. Al-Jobory, Ali Ismael

**Affiliations:** † Department of Physics, College of Science, Jouf University, Sakaka 72388, Saudi Arabia; ‡ Department of Physics, College of Science, University of Bisha, P.O. Box 551, Bisha 61922, Saudi Arabia; § Department of Physics, College of Science and Humanities in Al-Kharj, Prince Sattam bin Abdulaziz University, Al-Kharj 11942, Saudi Arabia; ∥ Physics and Engineering Mathematics Department, Faculty of Electronic Engineering, Menoufia University, Menouf 32952, Egypt; ⊥ Department of Physics, College of Science, University of Anbar, Anbar 31001, Iraq; # Department of Physics, Lancaster University, Lancaster LA1 4YB, U.K.; ∇ Department of Physics, College of Education for Pure Science, Tikrit University, Tikrit 3400, Iraq

## Abstract

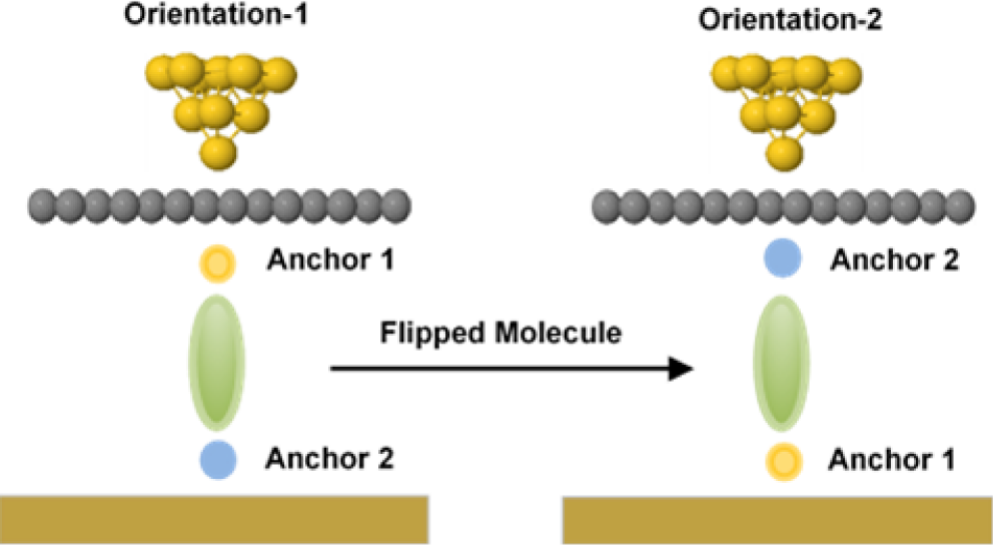

The orientational effect concept in a molecular-scale junction is established for asymmetric junctions, which requires the fulfillment of two conditions: (1) design of an asymmetric molecule with strong distinct terminal end groups and (2) construction of a doubly asymmetric junction by placing an asymmetric molecule in an asymmetric junction to form a multicomponent system such as Au/Zn-TPP+M/Au. Here, we demonstrate that molecular-scale junctions that satisfy the conditions of these effects can manifest Seebeck coefficients whose sign fluctuates depending on the orientation of the molecule within the asymmetric junction in a complete theoretical investigation. Three anthracene-based compounds are investigated in three different scenarios, one of which displays a bithermoelectric behavior due to the presence of strong anchor groups, including *pyridyl* and *thioacetate*. This bithermoelectricity demonstration implies that if molecules with alternating orientations can be placed between an asymmetric source and drain, they can be potentially utilized for increasing the thermovoltage in molecular-scale thermoelectric energy generators (TEGs).

## Introduction

The growing concern about CO_2_ emissions, global warming, and energy supplies over the past two decades has focused attention on alternative clean methods of power generation. Thermoelectric methods offer the benefit of a solid-state construction and allow the energy recovery solution to be readily adapted to the underlying process. Applications are as diverse as automotive, marine, aerospace, medical, and the Internet of things. Thermoelectric devices can also provide effective thermal management, including microelectronics and battery conditioning in electric vehicles and refrigeration in an all-solid-state device. Solid-state thermoelectric generators have been used effectively in niche applications, such as satellite missions for more than 50 years. There are now considerable opportunities to use thermoelectrics in a wide variety of domestic and industrial applications, including off-grid generation of electricity. However, to exploit thermoelectrics fully as energy harvesters in different environments requires the development of new thermoelectric materials with enhanced performance over wider temperature ranges, along with high-performance modules and systems.^1−6^ Most of the world’s power usage has recently been lost in the form of waste heat.^7−9^ Converting a low-quality form of energy to electricity has drawn the attention of several research groups. This energy is complicated to convert due to its molecular scale, which makes it difficult to characterize its structure.^10,11^ Single-molecule junctions and self-assembled monolayers (SAMs) are considered motivating alternatives for thermoelectric devices with definite atomic structures.^12−15^ Thus, studying charge transport and electrical and thermal properties can be controlled by three essential features: its anchor groups linked to electrodes, the quantum interference effect, and redox chemistry.^16−28^ These properties can be employed to various materials, including organic materials, such as porphyrins, due to their valuable applications.^29^

Porphyrins are organic chromophores known as macromolecular heterocyclic combinations consisting of porphin (C_20_H_14_N_4_) replaced by different functional groups at the *meso*-position or β-position. Free-base porphyrins can be doped with various metal ions at the porphyrin center to form metalloporphyrins.^30−33^ These porphyrins are obtainable in nature and are remarkable in organisms. Moreover, porphyrins possess several valuable properties, such as excellent thermal and chemical stability, also featuring photophysical and electrochemical properties due to the large π-aromatic system. These distinct properties can be controlled by exchange patterns on porphin and the coordinated metal ions at the porphyrin center. Thin films of porphyrin can be fabricated in various methods, including spin-coating,^31^ dip-coating,^34^ Langmuir–Blodgett,^35^ electropolymerization,^36^ thermoevaporation,^37^ and the self-assembled monolayer (SAM).^38−42^ The preferable method is the SAM technique, which can fabricate an ultrathin film with a well-controlled structure. Also, the SAM contains excellent thermal and mechanical stability. We previously investigated multicomponent SAMs^33,43−45^ as a complex structure of a Zn-TPP (zinc-tetraphenylporphyrin) and graphene (Gr) sheet with an anthracene molecule placed between two gold electrodes as a new strategy for the design of thin-film thermoelectric materials. They obtained good agreement between experimental and theoretical studies. They also proved that the SAMs represented high-quality monolayers that could enhance Seebeck coefficients. Furthermore, our group^46^ and others also explored the effect of different terminal groups on the Seebeck coefficients and conductance^19,47−49^ through design of the asymmetric anthracene molecule linked to the Gr sheet and placement between two gold electrodes. They reported that asymmetric anthracene–based molecules contain three different terminal groups, such as pyridyl, thioacetate, and SnMe_3_. They found that anthracene-based molecules with a thioacetate terminal group at one end and a pyridyl terminal group at the other end exhibit bithermoelectric behavior. Thus, a change in the orientation of the molecule (flipping the molecule horizontally at 180 °) would lead to a change in the sign of the Seebeck coefficients. These studies can be extended to different materials as a multicomponent system for electronic and thermal studies of the generation of thermopower.

**Figure 1:**
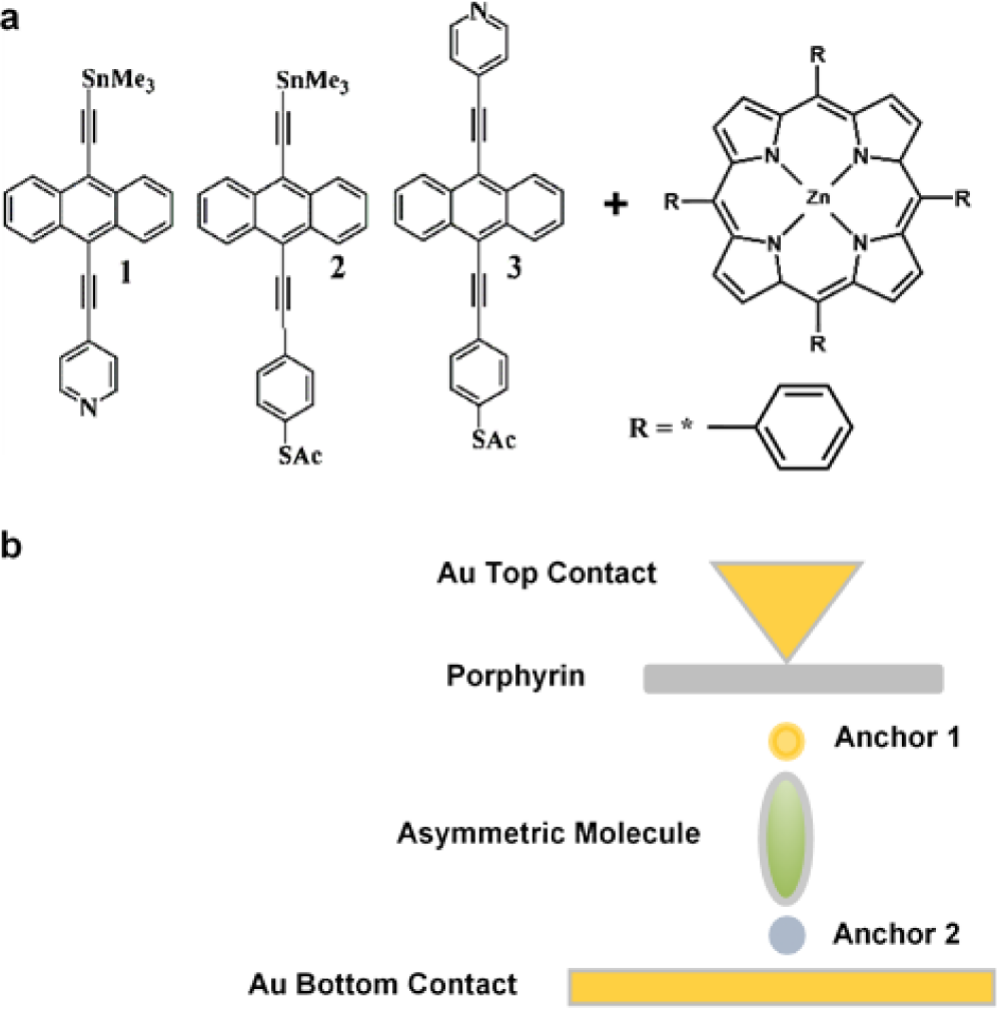
**(a**) Chemical structures of a multicomponent involving molecules **1–3** plus a porphyrin layer. (**b**) Typical schematic of a fabricated junction.

In the current study, we investigated the electronic properties of 3 asymmetric molecules (Figure 1a). The major considerations here are dedicated to the electronic structure properties, including frontier orbitals, optimization, and energy difference. These parameters have an essential influence on the electric and thermoelectric transport of the studied junctions. This work primarily focuses on the flipping characteristic in nanoscale junctions. To explore the flipping concept, three asymmetric anthracene-based molecules were employed and then combined to a porphyrin layer to form a multicomponent system, such as Au/Zn-TPP+M/Au (Figure 1b). In the presence of the porphyrin layer, asymmetric molecules could flip their orientations to provide two different geometries. These two geometries yield two different signs of the Seebeck coefficient.

## Results and Discussion

### Transmission Coefficient

The thermoelectric properties of 9 asymmetric junctions were explored using a combination of density functional theory and quantum transport theory to obtain the transmission coefficient ***T***(***E***), describing electrons of energy ***E*** passing from the source to the drain electrodes. From this, the room temperature electrical conductance ***G*** and Seebeck coefficient ***S*** were calculated as a result. We used a double-ζ plus polarization orbital basis set, norm-conserving pseudopotentials, the local density approximation (LDA) exchange-correlation functional, and an energy cutoff of 250 Rydbergs to define the real space grid. We also computed the results using the generalized gradient approximation (GGA) of the exchange and correlation functional used with the Perdew–Burke–Ernzerhof parametrization (PBE)^50^ and found that the resulting transmission functions were comparable^51−53^ to those obtained using LDA.

To examine these properties, the single-molecule junctions were studied in gold-gold junctions. As a first step, we designed asymmetric anthracene-based molecules **1, 2**, and **3**, such as the ones shown in Figure 1a. The optimum geometries of the isolated molecules were obtained by relaxing the molecules until all forces on the atoms were less than 0.01 eV/Å, as shown in Figure S1. As a first step toward understanding their electronic properties, the frontier orbitals of the asymmetric molecules **1–3** were computed. The highest-occupied molecular orbitals (HOMO) and lowest-unoccupied orbitals (LUMO), as well as the (HOMO–1) and (LUMO+1) of the studied molecules, have more weight on certain anchors, such as SAc and pyridyl compared to SnMe, as shown in Figures S2–S4. Next, the optimum distance between the three asymmetric molecules and the Au electrode was calculated. These asymmetric molecules have different anchor groups, including pyridyl, thiol, and SnMe3 (see Figures S5–S7). It should be noted that for both SAc and SnMe3 anchor groups of molecules **1–3** (Figure 1a), some changes occur when SAc and SnMe3 groups attach to a gold surface. In particular, the SAc group cleaves to form an S-Au bond.^54^ Similarly, SnMe3 also cleaves to form a direct C-Au bond.^54−56^ Using a combination of density functional theory (DFT) and the counterpoise method, the binding energies (BE) and optimum distances (*d*_Anch_) were calculated. More details, are summarized in the binding energy section in Table S1. After constructing the Au/molecule/Au junction, the transmission coefficient *T*(*E*) was calculated, for **1–3,** in three different cases. For more details, see Section 3 in the Supporting Information.

Figure S8 (right panel) illustrates the optimized junction of Au/**1**/Au, after the SnMe3 group cleaves and forms a C-Au direct bond. One would expect this molecule to be LUMO dominated if both anchors are pyridyl (symmetric). Nevertheless, the case is still true even if the molecule is asymmetric (i.e., Py and SnMe3), as shown in the left panel of Figure S8. We believe this is due to the Py anchor overcoming the SnMe3 (Au-C), even though the binding energy of SnMe3 is approximately twice as strong as that of Py, as summarized in Table S1 (−1.0 versus −0.45 eV). It is worth mentioning that some studies^57^ demonstrate that SnMe3 is a HOMO-dominated anchor. On the contrary, Figure S9 demonstrates that Au/**2**/Au junction possesses a HOMO-dominated transport. The DFT-predicted Fermi energy  sits close to the HOMO resonance because both anchors (thiol and SnMe3) are pinning in the same direction toward the HOMO resonance. While Au/**3**/Au junctions include thiol and pyridine and these anchors are well-known to pin down in an opposite direction, in other words, HOMO dominated or LUMO dominated. Furthermore, both anchors are widely accepted to be strong anchors; as a result, one would expect molecule **3** to possess midgap transport (i.e., Fermi energy ), rather than a HOMO or LUMO, as illustrated in Figure S10. Despite the fact that **1–3** are asymmetric molecules and undergo cleavage, however, the asymmetric molecule cannot flip in the junction as the top and bottom electrodes are identical Au/M/Au. More computational details are provided in the Supporting Information.

**Figure 2:**
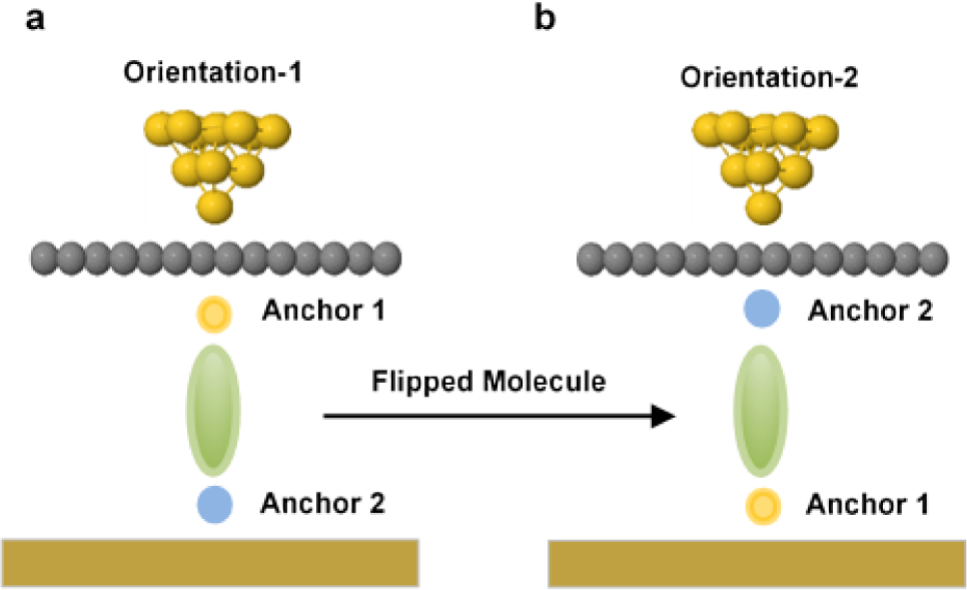
Schematic illustration of molecular junctions with two orientations. Orientation-1 and -2 show how the molecule flips between the porphyrin layer and Au substrate (**a** and **b,** respectively).

To establish the orientational effects in a junction, there are two conditions that need to be satisfied: (1) an asymmetric molecule, such as the ones (**1–3**) shown in Figure 1a and (2) An asymmetric junction. To do so, we inserted an extra segment to the Au/M/Au junction, which is a zinc-tetraphenylporphyrin (Zn-TPP) to form the multicomponent Au/Zn-TPP+M/Au (Figure 1b). Experimentally, this means Zn-TPP–coated gold contact. More details about the synthetic and STM measurements for similar junctions can be obtained from ref. (43). In the present research, the Zn-TPP is stationary, while the asymmetric molecule flips between two orientations, as shown in Figure 2.

For the flipping purpose, we explored 3 scenarios: a, b, and c. Molecule **1** has been nominated for scenario a. Figure S15 illustrates the components that are used to build the flipping junction (**1** consists of SnMe3 and pyridyl terminal end groups). It should be kept in mind that the SnMe3 anchor does not experience cleavage during the flipping procedure when attached to the Zn-TPP layer, unlike in a symmetric junction (Au/M/Au). The same simulations were repeated to calculate the transmission coefficient *T*(*E*), as described in the Au/M/Au junctions above. The two *T*(*E*) curves demonstrate a LUMO-dominated transport. However, the DFT-predicted Fermi energy () location in the HOMO–LUMO gap varies from one orientation to another. Specifically, orientation-2 is slightly more toward the low energy (i.e., midgap), as illustrated in Figure S16.

In scenario b, the same procedure that was applied in scenario a was repeated; however, with different terminal end groups. This scenario mainly included SnMe3 and SAc. Here, both anchors (i.e., SnMe3 and SAc) did not experience cleavage when attached to Zn-TPP layer, unlike SAc with an Au surface, as shown in Figure S17. The two transmission function curves are slightly biased toward the HOMO resonance; however, they differ by the Fermi energy  position, as shown in Figure S18. Scenario b exhibits an opposite behavior to scenario a (i.e., LUMO and HOMO dominated).^58−61^ We attribute the origin of this trend to the fact that the pyridine anchor pins toward the LUMO resonance, whereas the thiol pins toward the HOMO resonance. As a result of this, we conclude that even though the molecule possesses two different anchor groups, the strongest anchor drives the transport type of the whole scenario (i.e., HOMO or LUMO). For example, scenario a is LUMO dominated due to the presence of a pyridine anchor, while scenario b is HOMO-dominated due to the presence of a thiol anchor (pyridine and thiol are known to be strong anchors compared to SnMe3).

**Figure 3:**
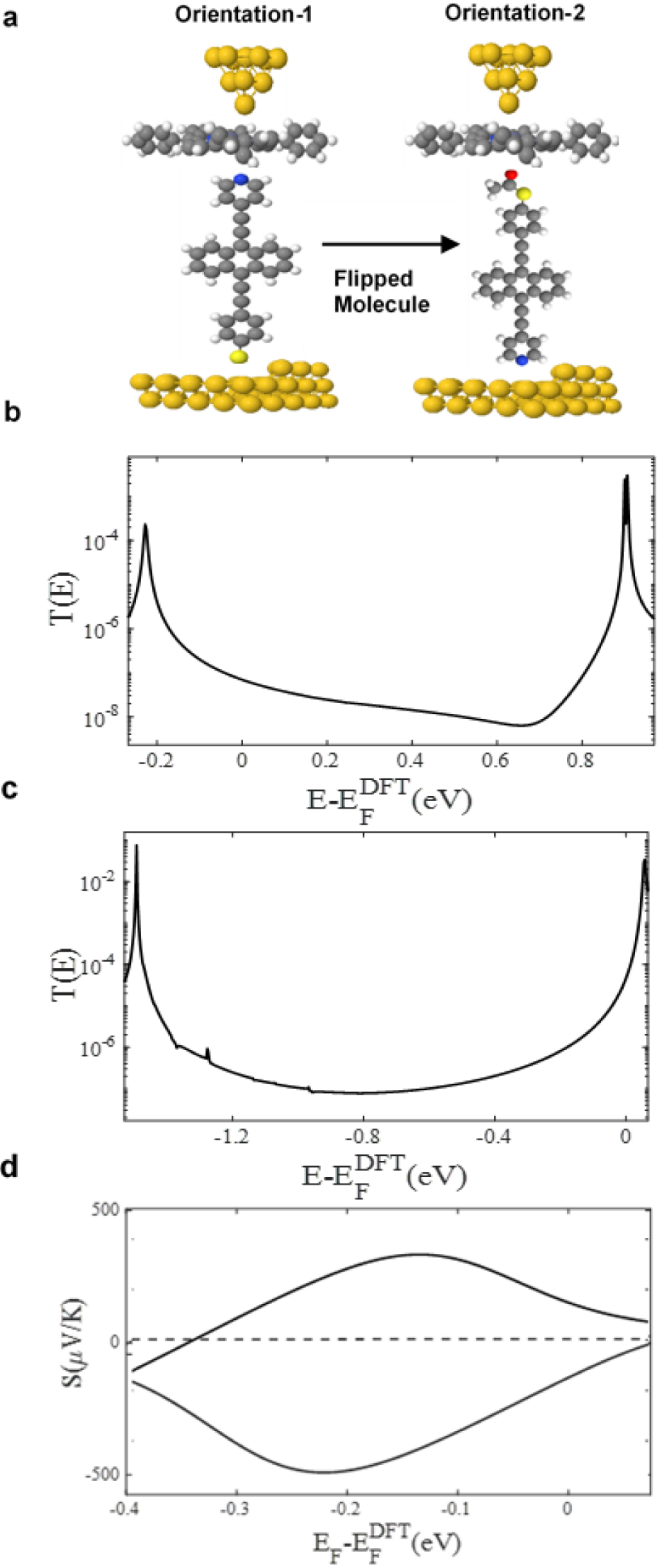
**(a**) Schematic illustration of molecular junctions of two orientations. Orientation-1 and -2 show how the molecule flips between the porphyrin layer and the Au substrate. Orientation-1 is when *Py* is linked to the porphyrin from one end and *S* to Au from the other end. Orientation-2 is the opposite; *SAc* linked to the porphyrin and *Py* to the Au contact. (**b,c**) Zero-bias transmission coefficients *T(E)* against electron energy *E*. The flipping characteristic switches the Fermi energy (E-*E*_*F*_^DFT^ = 0 eV), from LUMO-resonance toward HOMO-resonance. (**d**) Seebeck coefficient *S* as a function of the energy of orientation-1 and -2. Orientation-1 exhibits a positive *S*, whereas orientation-2 shows a negative *S*.

Now the question is what happens when a scenario involves two different anchors but both are strong and pin down in an opposite direction, such as pyridine and thiol; scenario c shall investigate this case. Figure 3a displays the flipping orientations of asymmetric molecules with strong anchor groups. It should be noted that the thioacetate group (SAc) cleaves during the flipping procedure, as illustrated in the left panel of Figure 3a (orientation-1).

Figure 3b,c, illustrates the transmission coefficient curves for orientation-1 and -2 of molecule **3**. Panel b demonstrates a HOMO-dominated transport as the Fermi energy  is closer to the HOMO resonance. On the contrary, panel c exhibits a LUMO-dominated transport as the Fermi energy  is within the vicinity of HOMO resonance. This disparity can be explained by the opposite behavior of the two different anchor groups within an asymmetric junction (i.e., Au/Zn-TPP+M/Au). This contrasted behavior can be revoked if the porphyrin layer pulls out of the junction and ends up as a symmetric system (Au/M/Au), such as case 3 in Figures S10 and S13.

### Seebeck Coefficient

After computing the electronic transmission coefficients for the symmetric and asymmetric junctions, including Au/**1–3**/Au and Au/Zn-TPP+**1–3**/Au, we shall now compute their Seebeck coefficients. To this end, it is useful to introduce the non-normalized probability distribution *P*(*E*), defined by:^62^
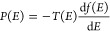
1where *f*(*E*) is the Fermi function and *T*(*E*) is the transmission coefficient, whose moments *Li* are denoted as follows:
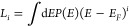
2where *E*_*F*_ is the Fermi energy. The Seebeck coefficient *S* is then given by:
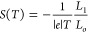
3where *e* is the electronic charge.

It is noteworthy that the slope of the transmission coefficient *T*(*E*) determines the sign and magnitude of the Seebeck coefficient *S*; in other words, whether the curve is HOMO or LUMO dominated. To begin with the symmetry junctions, Figure S11 displays a negative Seebeck coefficient at the DFT-predicted Fermi eV, and this is due to the fact that molecule **1** is LUMO dominated, as shown in Figure S8.

In contrast, Figure S12 presents a positive *S* at  eV because molecule **2** is a HOMO-dominated molecule, as shown in Figure S9. While Figure S13 shows a negative *S* at  eV, again because molecule **3** is slightly a LUMO-dominated molecule at the Fermi energy, as shown in Figure S10.

The next step was to determine *S* for asymmetric junctions as the insertion of a Zn-TPP layer generates two configurations (i.e., flipping simulations). For scenario a, as the two orientations were LUMO-dominated curves (Figure S16, top panel), this should reflect in the Seebeck sign, which means the two curves possess negative Seebeck coefficients, as illustrated in the lower panel of Figure S16. Similar simulations were repeated for scenario b, and since both transmission functions are HOMO curves (Figure S18, top panel), this leads to positive Seebeck coefficients, as shown in the lower panel of Figure S18. Both scenarios a and b deal with the case when the two orientations exhibit one type of transport, either LUMO or HOMO, and this is not the case in scenario c. Scenario c comprises an asymmetric molecule and is sandwiched by an asymmetric junction (Au/Zn-TPP-**3**/Au). Furthermore, the two anchors of this molecule are strong; however, they pin down in different directions. The recipe of the Au/Zn-TPP-**3**/Au junction satisfies the flipping characteristic; as a result, Figure 3d displays both the positive and negative Seebeck coefficients. We attribute this bithermoelectric behavior to the effect of inserting the porphyrin layer in a symmetric junction since in the absence of the Zn-TPP layer, no such sign change occurs. Indeed, these results show that the top porphyrin-coated contact defines the transport type, with Au+ Zn-TPP-Py or Au+ Zn-TPP-SAc being either LUMO- or HOMO-dominated transport, respectively. It should be noted that the location of Fermi level  is essential in this study as it determines whether the electronic transport is dominated by holes or electrons (i.e., HOMO or LUMO), as shown in Figures S8S12–. Furthermore, HOMO or LUMO transport reflects in the sign of the Seebeck coefficient, which is the base of the flipping concept.

Table 1 summarizes the results of studying 3 asymmetric molecules: first, in symmetric junctions (Au-Au), when only one orientation is present, and then in asymmetric junction gold-porphyrin (Au-Po), while two orientations occur and lead to positive and negative Seebeck coefficients. The occurence of two different signs was due to attachment of the Au electrode to different types of anchors, such as thiol, pyridyl, and TMS.

**Table 1:** Studied Molecules **1-3**, in Symmetric Junction Au-Au (One Orientation, No Flipping) and in Asymmetric Junction Gold-Porphyrin Au-Po (Two Orientations)^a^

molecule	junction	orientation	*S* sign	cause
**1**	Au–Au	one	–	pyridyl anchor
**2**	Au–Au	one	+	thiol anchor
**3**	Au–Au	one	–	thiol anchor
**1**	Au–Po	1 2	– –	TMS anchor pyridyl anchor
**2**	Au–Po	1 2	+ +	TMS anchor thiol anchor
**3**	Au–Po	1 2	+ –	thiol anchor pyridyl anchor

a Flipping occurs in asymmetric junctions and casing the Seebeck coefficient to switch sign (+, −) due to attachment to different anchor groups, including thiol, pyridyl, and TMS.

## Conclusions

We have established the orientational effects in a molecular junction, and this is required to fulfill two conditions: (1) design an asymmetric molecule, such as **1–3**, as shown in Figure 1a and (2) originate doubly an asymmetric junction, by placing an asymmetric molecule in an asymmetric junction to form a multicomponent system, such as Au/Zn-TPP+M/Au (Figure 1b). This work has examined asymmetric systems that are capable of switching the sign and enhancing the Seebeck coefficients of asymmetric junctions. In the presence of a porphyrin top contact, we conclude that flipping the orientations of molecules **1** and **2** changes the magnitude; however, not the sign of the Seebeck coefficient, whereas molecule **3** is discovered to be bithermoelectric, manifesting Seebeck coefficients of either sign depending on its orientation within the doubly asymmetric junction.

As reported by X-ray photoelectron spectroscopy (XPS), measurements of similar systems, sandwiching asymmetric molecules, such as **1**, **2,** and **3**, in Au/Gr+M/Au junctions will lead to both possible orientations and will produce molecular films with either a positive or negative Seebeck characteristic. STM measurements of a single-molecule’s Seebeck coefficient for **1** and **2** would fluctuate in magnitude; however, not in sign, across the film. In contrast, for SAMs formed from **3**, single-molecule STM-based measurements would yield values of *S*, with random signs across the film. These qualitatively featured behaviors provide new insights into the thermoelectric properties of SAMs. They also explain that in the case of **3**, if the orientations of molecules in neighboring islands could be controlled, to create SAMs with alternating orientations and therefore Seebeck coefficients of alternating signs, then these could form a basis for improving the thermovoltage in nanoscale thermoelectric generators.

## Supporting Information Available

Theoretical details, binding energy, transmission coefficient ***T***(***E***), Seebeck coefficient ***S***, flipping characteristic (PDF)
