# Supplementary material for: Orientational Effects and Molecular-Scale Thermoelectricity Control
Source: ACS Omega. 2024 Jun 26;9(27):29537–43. doi: 10.1021/acsomega.4c02141 (PMC11238236; doi:10.1021/acsomega.4c02141)
Supplement: Supplementary file 1 — ao4c02141_si_001.pdf [file ao4c02141_si_001.pdf]

# Supporting Information

## Orientational effects and molecular-scale thermoelectricity control

Turki Alotaibi,[‡,a] Maryam Alshahrani,[‡,b] Majed Alshammari,[‡,a] Moteb Alotaibi,[‡,c] Taha Abdel Mohaymen Taha,[d] Alaa A. Al-Jobory,[e] and Ali Ismael,[f, g*]

*Department of Physics, College of Science, Jouf University, Sakaka, Saudi Arabia.*
*Physics Department, LancasterUniversity, Lancaster LA1 4YB, U.K.; Physics Department, College of Science, University of Bisha, Bisha 61922, Kingdom of Saudi Arabia.*
*Department of Physics, College of Science and Humanities in Al-Kharj, Prince Sattam bin Abdulaziz University, Al-Kharj 11942, Saudi Arabia.*
*Physics and Engineering Mathematics Department, Faculty of Electronic Engineering, Menoufia University, Menouf 32952, Egypt.*
*Department of Physics, College of Science, University of Anbar, Anbar, Iraq.*
*Department of Physics, Lancaster University, Lancaster LA1 4YB, UK.*
*Department of Physics, College of Education for Pure Science, Tikrit University, Tikrit, Iraq.* *‡ These authors contributed equally to this work.*

## Table of contents

**1. Theoretical details**

**2. Binding energy**

**3.Transmission coefficient $T(E)$**

**4.Seebeck coefficient $S$**

**5. Flipping characteristic**

**6. References**

## 1. Theoretical details

### 1.1 Optimised DFT Structures of Isolated Molecules

Using the density functional theory (DFT) code,[1, 2] the optimum geometries of the isolated molecules **1-3**, and ZnTPP were obtained by relaxing the molecules until all forces on the atoms were less than 0.01 eV / Å as shown in Fig. SI.1. A double-zeta plus polarization orbital basis set, norm-conserving pseudopotentials, with an energy cut-off of 250 Rydbergs, defined on the real space grid was used and the generalized gradient approximation (GGA) was chosen to be the exchange correlation functional.

**a**

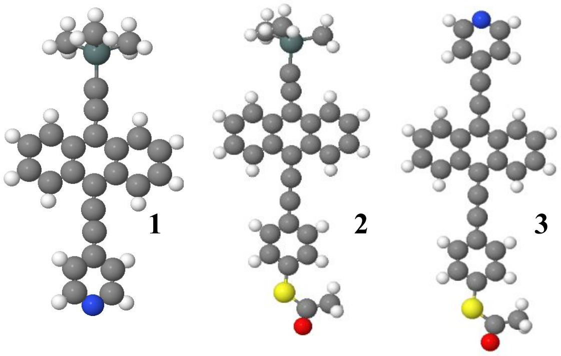


**Figure SI.1: a**: Asymmetric anthracene-based molecules. **1**: Anthracene with *$SnMe_3$* and *Py* anchors, **2**: Anthracene with *$SnMe_3$* and *SAc* anchors, and **3**: Anthracene with *Py* and *SAc* anchors.

Figure SI.1 shows three structures of asymmetric anthracene-based molecules. These structures are fully relaxed, and are as follows, **1**: anthracene-based molecule with two different anchors including *$SnMe_3$*

and pyridine (*SnMe*$_3$-anthracene-*Py*), **2**: anthracene-based molecule with *SnMe*$_3$ and thioacetate (*SnMe*$_3$-anthracene-*SAc*), **3**: anthracene-based molecule with pyridine and thioacetate (*Py*-anthracene-*SAc)*.

### 1.2 Frontier orbitals of the molecules.

In this section, we show the frontier orbitals of the studied molecules: highest occupied molecular orbitals (HOMO) and lowest unoccupied orbitals (LUMO), in addition to (HOMO-1), and (LUMO+1), along with their energies.

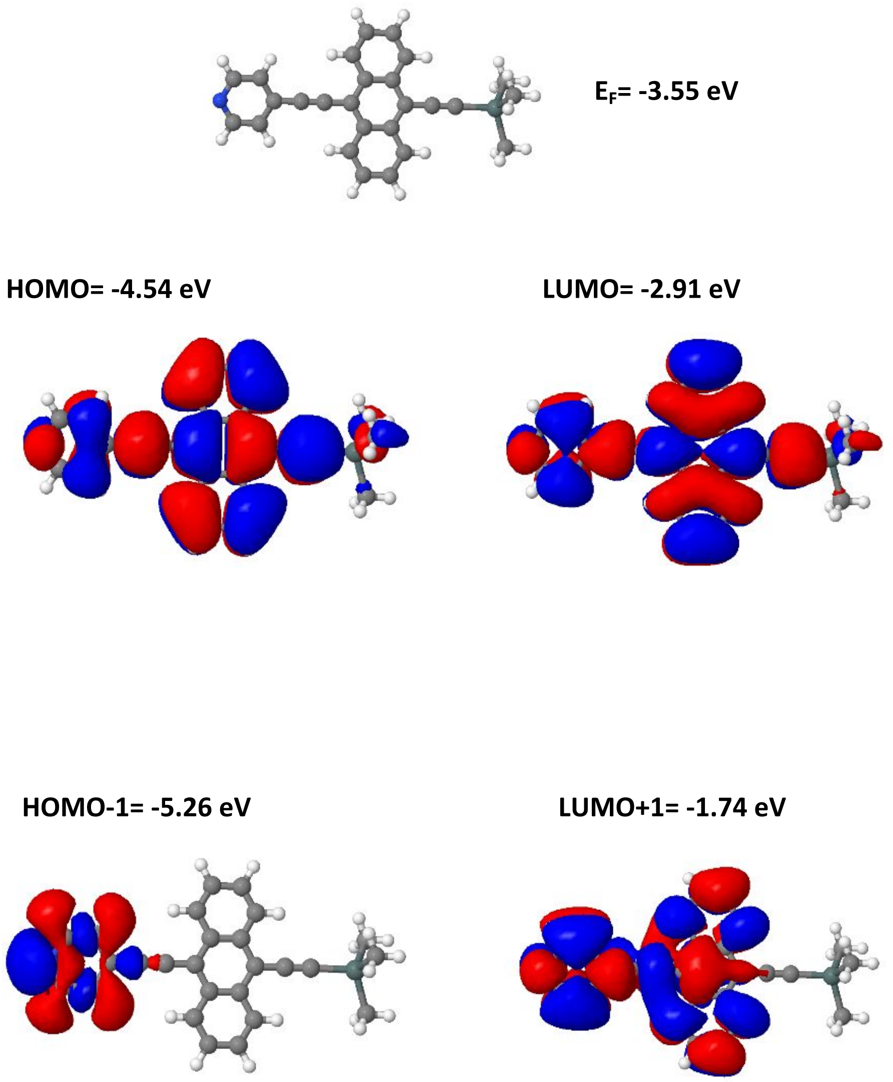


**Figure SI.2:** Wave function for **1**. Top panel: fully optimised geometry of **1**. Lower panel: HOMO, LUMO, HOMO-1, LUMO+1 of molecule **1** along with their energies.

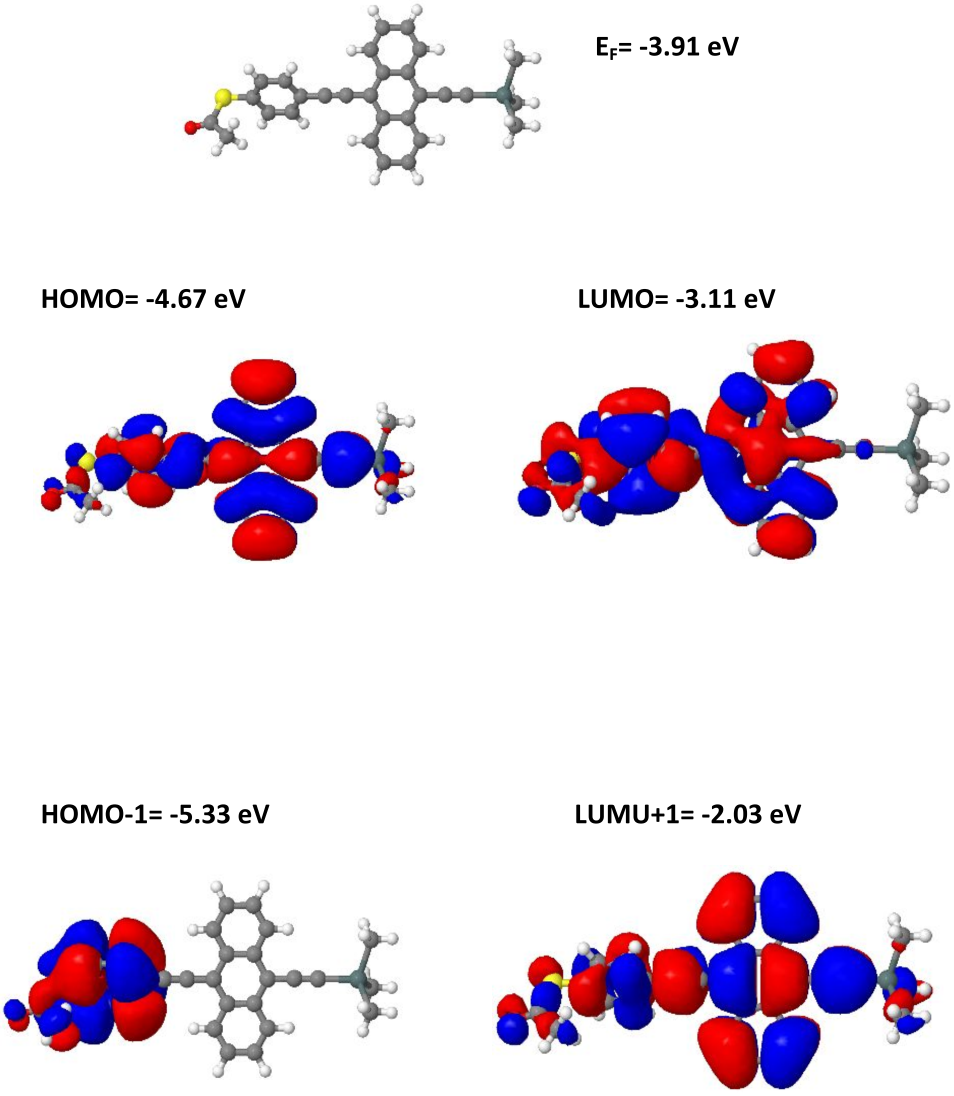


**Figure SI.3:** Wave function for **2**. Top panel: fully optimised geometry of **2**. Lower panel: HOMO, LUMO, HOMO-1, LUMO+1 of molecule **2** along with their energies.

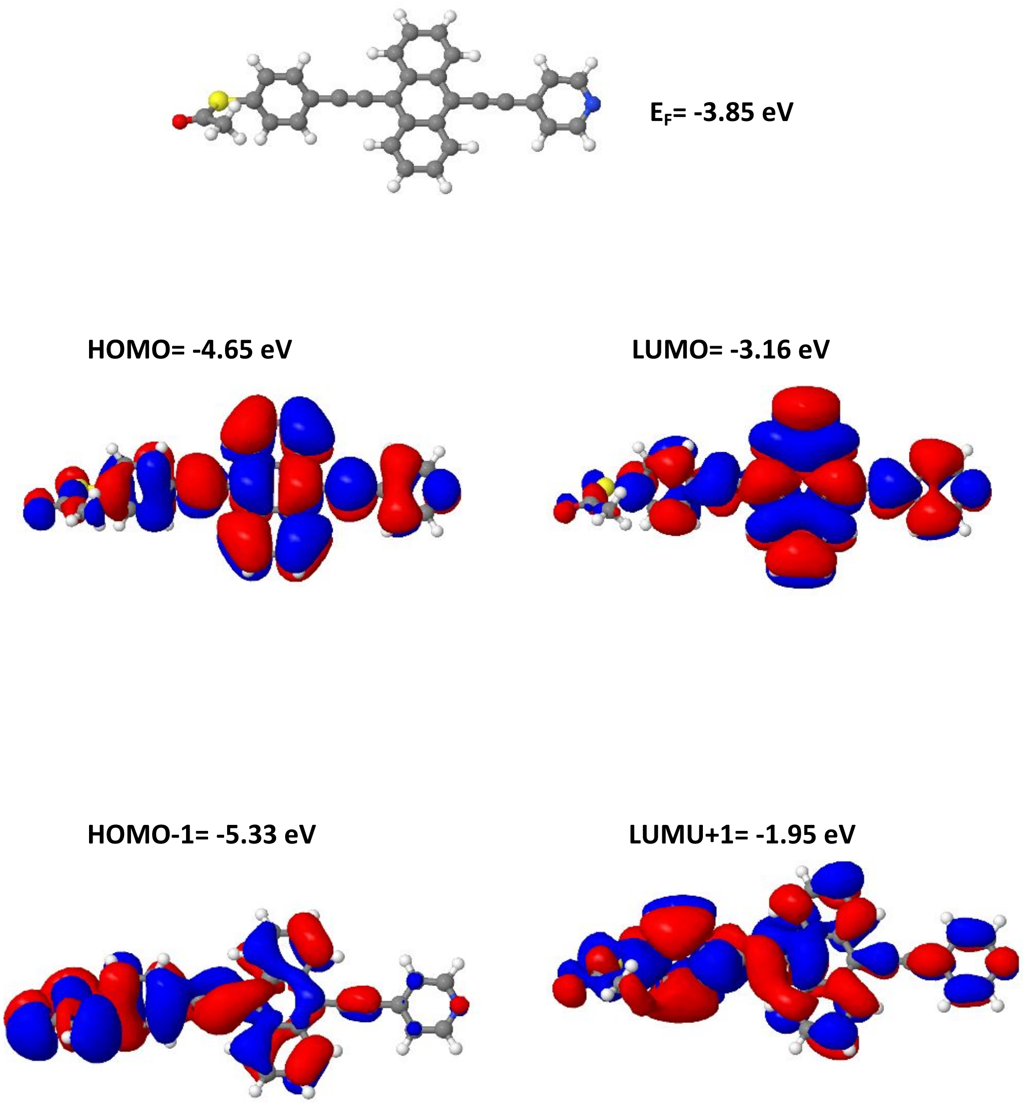


**Figure SI.4:** Wave function for **3**. Top panel: fully optimised geometry of **3**. Lower panel: HOMO, LUMO, HOMO-1, LUMO+1 of molecule **3** along with their energies.

## 2. Binding Energy

This section uses a combination of DFT and the counterpoise method. Briefly, the latter removes the basis set superposition errors when calculating the optimum binding distance of two objects; for more details see[3, 4] .

### 2.1 Binding Energy of Anthracene Core to Gold substrate:

Here, we calculated the binding energy of the anthracene-based molecules as shown in Figs. SI 5-7, on a gold substrate. These asymmetric molecules have different anchor groups including: *Py*, thiol, and *TMS* (see Figs. SI 5-7). It should be noted that for both *SAc* and *SnMe3* groups of molecules **1-3** (see

Fig. 1a in the manuscript), some changes occur when *SAc* and *SnMe3* groups attach to a gold metal. In particular, the *SAc* group cleaves to form a *S-Au* bond [5]. Similarly, *SnMe3* cleaves to form a direct *C-Au* bond[5].

Figure SI.5 (A1), shows that the optimum binding distance $d_{Anch}$. between the *Py* anchor and the *Au* to be 2.3 Å, and at approximately -0.4 eV. It is worth mentioning that in this case there the molecule remains as it is, meaning no changes as shown in Figure 1a (molecule **3**), in the manuscript.

Similarly, Figure SI.6 (A2) represents the binding energy between the thiol anchor group and the gold lead and $d_{Anch.}$ is 2.4 Å, at approximately -1.2 eV. This suggests the binding energy of the thiol anchor group is much stronger than that the *Py* anchor to *Au* electrode (compare Fig. SI.6 against Fig. SI.5). This result in agreement with the literature review, it should be noted that the *SAc* group cleaves when this group brought close to the *Au* metal to form *S-Au* bond.

Figure SI.7 (A3) exhibits the binding energy between the *TMS* anchor group and the gold lead. The TMS's (*Au-C*), binding energy lies between the *S* and *Py*, however, it is more towards the stronger binding energy (i.e. thiol) to Au with binding energy of -1 eV at $d_{Anch.}$ = 2.3 Å. These calculations suggest that both thiol and *TMS* bind to Au substrate approximately 3 times stronger than that *Py* anchor. Again, the SnMe3 group cleaves when this group brought close to the Au metal to form *C-Au* direct bond, (Note the optimum distance between the Au and Anchor labelled $d_{Anch.}$).

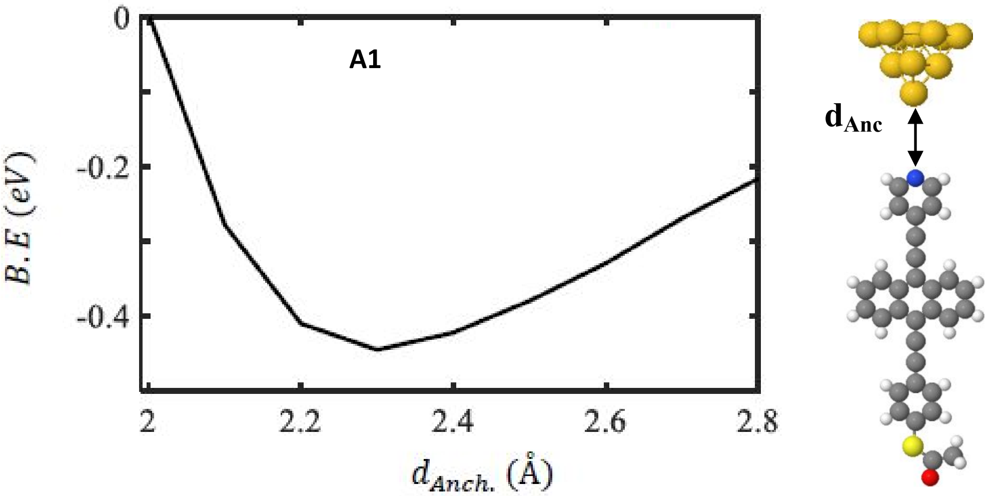


**Figure SI.5**: An asymmetric anthracene-based molecule configuration with thioacetate and pyridine anchors at the *Au* lead interface *Au-Py* (**right**). Binding energy as a function of the optimum binding distance $d_{Anch}$., where $d_{Anch}$. is found to be approximately 2.3 Å. Key: C = grey, H = white, S = light yellow, Au = dark yellow, N = blue, O = red.

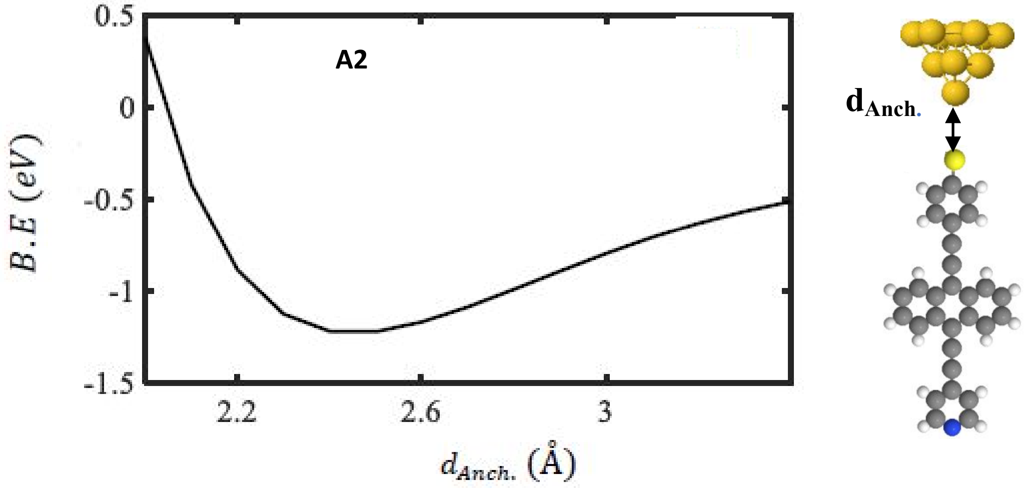


**Figure SI.6**: An asymmetric anthracene-based molecule configuration with thioacetate and pyridine anchors at the *Au* lead interface *Au-S* (**right**). Binding energy as a function of the optimum binding distance $d_{Anch.}$, where $d_{Anch.}$ is found to be approximately 2.4 Å. Key: C = grey, H = white, S = light yellow, Au = dark yellow, N = blue.

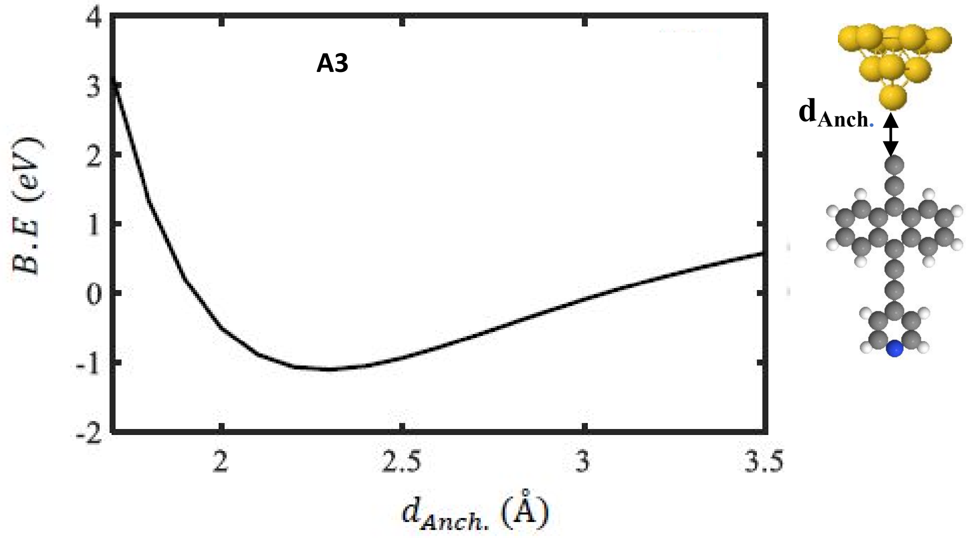


**Figure SI.7**: An asymmetric anthracene-based molecule configuration with thioacetate and pyridine anchors at the *Au* lead interface *Au-S* (**right**). Binding energy as a function of the optimum binding distance $d_{Anch.}$, where $d_{Anch.}$ is found to be approximately 2.2 Å. Key: C = grey, H = white, Au = dark yellow.

**Table S1:** Summarises all the binding energies (B.E), and optimum distances ($d_{Anch.}$ $and$ $d$), calculations for three different anchor groups bind to *Au.*

| Mol. | $d_{Anch.}$ (Å) | B.E (eV) |
|---|---|---|
| A1 | 2.3 | -0.45 |
| A2 | 2.4 | -1.2 |
| A3 | 2.2 | -1.0 |

## 3. Transmission coefficient $T(E)$

This section investigates the transmission function of asymmetric anthracene-based core molecules with different anchor groups including *SnMe3, Py* and *SAc* for this purpose we shall explore three different cases:

### 3.1 Case 1: Anthracene-based of *SnMe3* and *Py* anchor groups:

Anthracene molecule with two different anchors including *SnMe3* and *Py*, has been studied as shown in Figure SI.8. If the two anchors were pyridine, one would expect this molecule to be a LUMO-dominated due to the presence of the pyridyl anchor. However, it seems the case is still true even if the molecule is asymmetric, which means two different anchors. We believe this is due to that the *Py* anchor overcomes the *TMS* (*Au-C*), even though the binding energy of *TMS* is stronger than that *Py*. It is worth mentioning that, some studies[6] demonstrate that *TMS* is a HOMO-dominated anchor and that is clearly shown in Figure SI.11, where the *TMS* pulls the DFT-predict Fermi energy ($E - E_F^{DFT} = 0$ eV) slightly away from LUMO resonance, as the pyridyl anchor is pinning the Fermi level $E - E_F^{DFT} = 0\ eV$ so close to the LUMO resonance[7, 8]. It should be noted that the SnMe3 group cleaves when it attaches to Au contact to form *Au-C* direct contact, as we discussed that above.

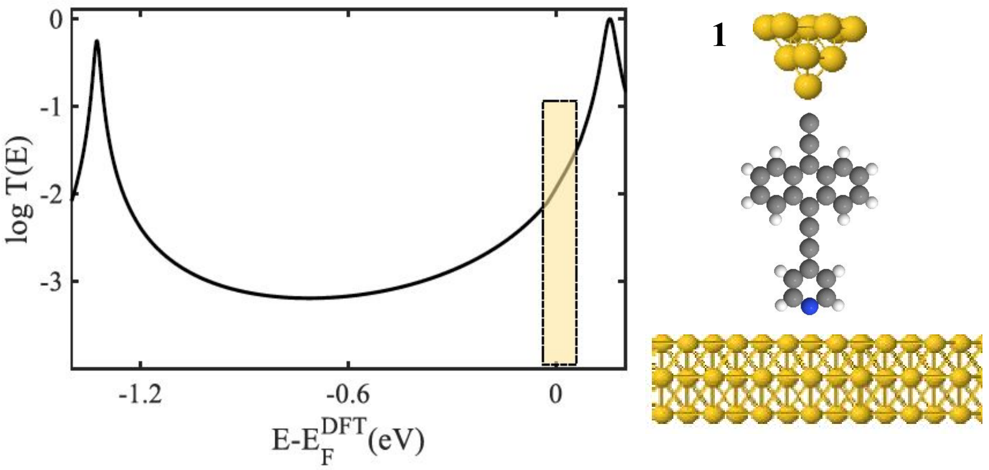


**Figure SI.8: Right panel:** Schematic illustrations of an asymmetric molecular junction of **1. Left panel:** Zero-bias transmission coefficient $T(E)$ of molecule **1** against electron energy E.

**3.2 Case 2: Anthracene-based of *SnMe3* and *SAc* anchor groups:**

In this case, we consider anthracene with two different anchors including *SnMe3* and *SAc* anchors. Figure SI.9 shows that this molecule is a HOMO-dominated and that is what one would expect due to the fact that the both anchors (*SnMe3* and *SAc*), are HOMO-dominated. The DFT-predicted Fermi energy $E - E_F^{DFT} = 0\ eV$ sits so close to the HOMO resonance because both anchors are pinning in the same direction toward HOMO resonance. Again, it should be noted that both anchors (*SnMe3* and *SAc),* cleave to end up with *Au-C* direct contact (tip side), and *Au-S* contact (substrate side).

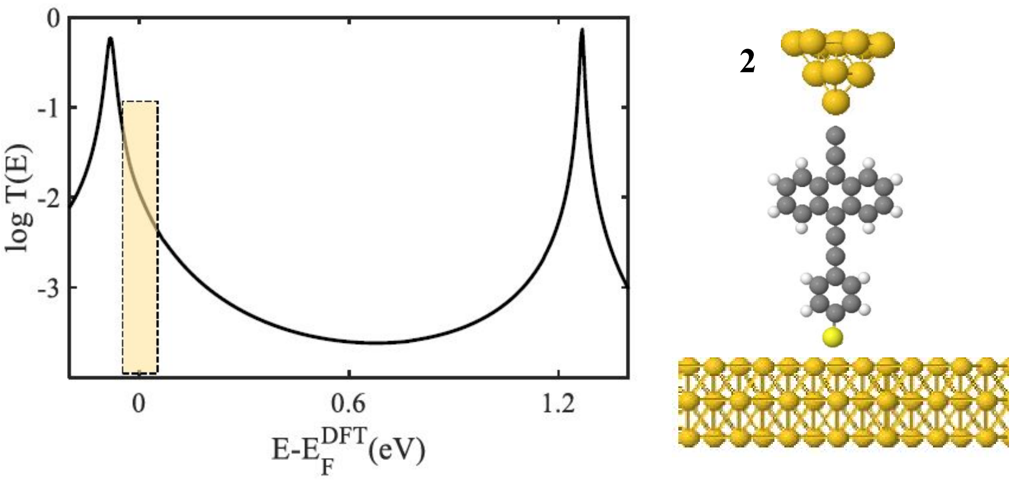


**Figure SI.9: Right panel:** Schematic illustrations of an asymmetric molecular junction of **2. Left panel:** Zero-bias transmission coefficient $T(E)$ of molecule **2** against electron energy E.

**3.3 Case 3: Anthracene-based of *Py* and *SAc* anchor groups:**

Case 3 is an asymmetric anthracene with two different anchors including thioacetate and pyridine, as shown in Figure SI.10. As the two anchors are well-known to pin down in an opposite direction, in other words, HOMO- or LUMO-dominated. Furthermore, both anchors are strong so one would expect this molecule to possess a mid-gap Fermi energy ($E - E_F^{DFT} = 0$ eV), rather than a HOMO or LUMO-dominated. Figure SI.10, proves this prediction to be accurate as clearly shown that Fermi energy locates in mid-way between the HOMO and LUMO resonances. Note: The cleavage procedure is happening at the *SAc* anchor and end up with *Au-S* contact.

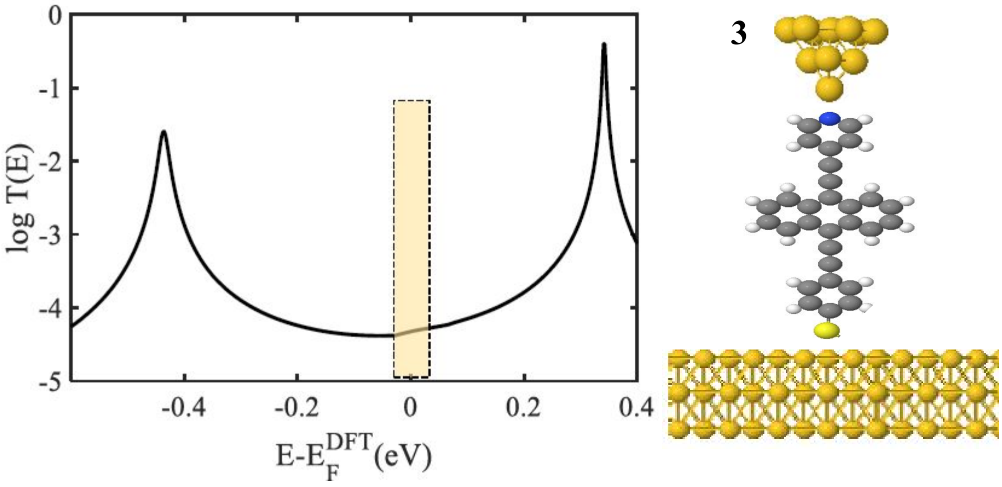


**Figure SI.10**: **Right panel**: Schematic illustrations of an asymmetric molecular junction of 3. **Left panel**: Zero-bias transmission coefficient $T(E)$ of molecule 3 against electron energy E.

## 4. Seebeck coefficient *S*

After computing the electronic transmission coefficient for the 3 junctions, we now compute their Seebeck coefficients $S$. To this end, it is useful to introduce the non-normalised probability distribution $P(E)$ defined by.

$$P(E) = -T(E)\frac{df(E)}{dE} \qquad \text{(S1)}$$

where $f(E)$ is the Fermi function and $T(E)$ is the transmission coefficients, whose moments $Li$ are denoted as follows

$$L_i = \int dE\, P(E)\,(E - E_F)^i \qquad \text{(S2)}$$

where, $E_F$ is the Fermi energy. The Seebeck coefficient, $S$ is then given by

$$S(T) = -\frac{1}{|e|T}\frac{L_1}{L_o} \qquad \text{(S3)}$$

where, $e$ is the electronic charge.

The slope of the transmission coefficient $T(E)$ determines the sign and magnitude of the Seebeck coefficient $S$. In other words, whether the curve is HOMO or LUMO dominated. Figure SI.14, shows a

negative Seebeck coefficient at the DFT-predicted Fermi $E - E_F^{DFT} = 0$ eV and this is due to the fact that molecule **1** is a LUMO-dominated as shown in Figure SI.8 (anthracene of *TMS* and *Py* anchors).

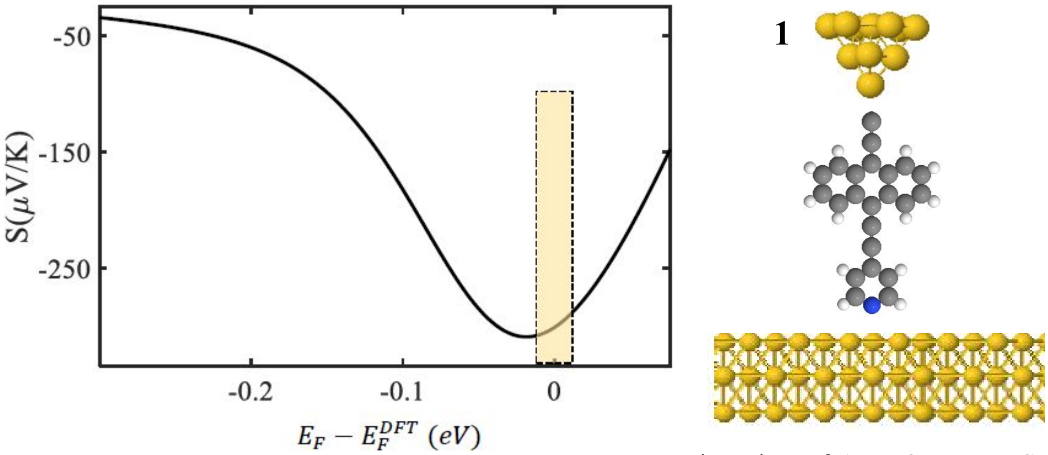


**Figure SI.11: Right panel:** Schematic illustrations of molecular junction of **1. Left panel:** Seebeck coefficient $S$ of molecule **1** against electron energy E.

In contrast, Figure SI.12 shows a positive $S$ at $E - E_F^{DFT} = 0$ eV, because molecule **2** is a HOMO-dominated molecule as shown in Figure SI.12 (anthracene of *TMS* and *S* anchors). Similarly, Figure SI.13 shows a negative $S$ at $E - E_F^{DFT} = 0$ eV, again because molecule **3** is slightly a LUMO- dominated molecule at the Fermi energy as shown in Figure SI.10 (anthracene of *Py* and *S* anchors).

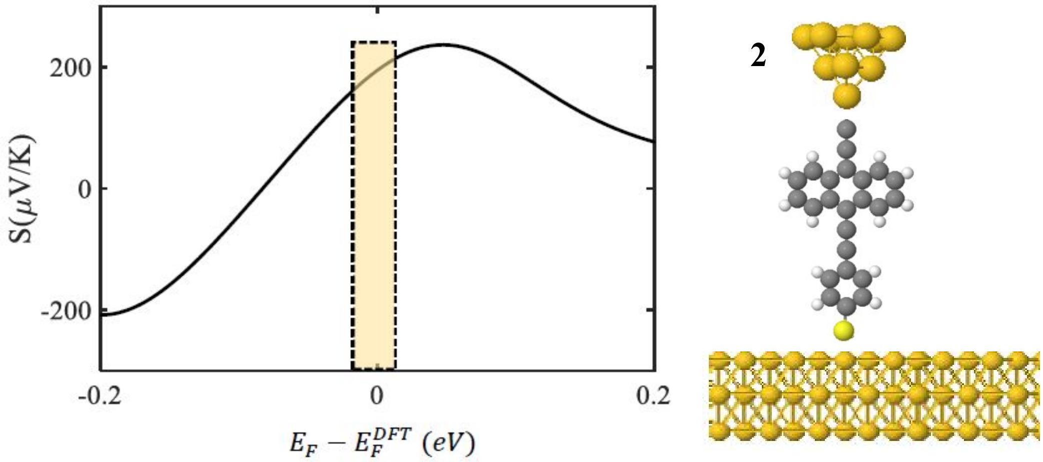


**Figure SI.12: Right panel:** Schematic illustrations of molecular junction of **2. Left panel:** Seebeck coefficient $S$ of molecule **2** against electron energy E.

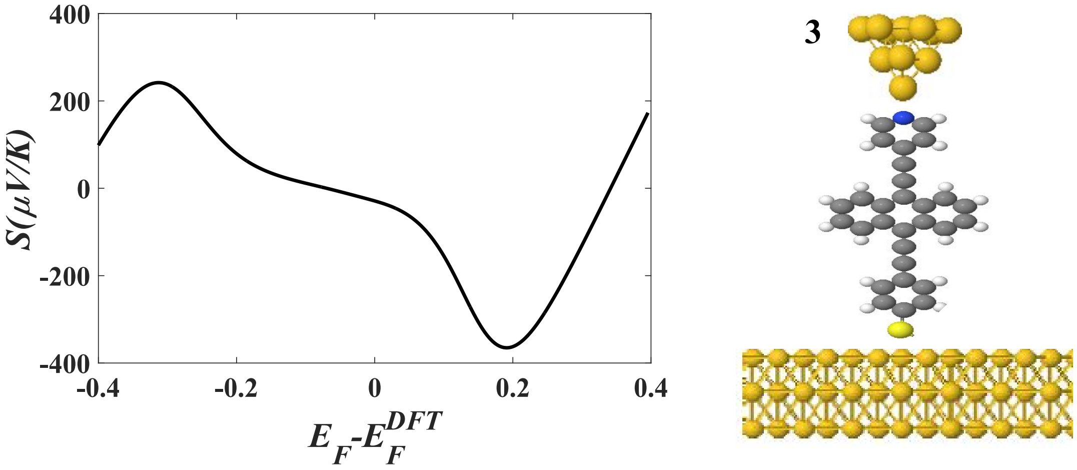


**Figure SI.13: Right panel:** Schematic illustrations of molecular junction of **3. Left panel:** Seebeck coefficient *S* of molecule **3** against electron energy *E*.

## 5. Flipping characteristic S

Following the simulations of 3 asymmetric anthracene-based molecules in *Au/M/Au* junctions. In this section we shall add an extra segment to the Au/M/Au junction, which is a *zinc-tetraphenylporphyrin (Zn-TPP)* as shown in Fig. SI.14b, to form multicomponent *Au/Zn-TPP+M/Au*. Experimentally, this means *Zn-TPP* coated gold contact for more detail about the synthetic and STM measurements we guide the reader to[9]. In the present research, the *Zn-TPP* is stationary, while the asymmetric molecule flips between two orientations, as shown in Figure 2a of the manuscript. For the flipping purpose, we shall investigate 3 scenarios **a**, **b** and **c**.

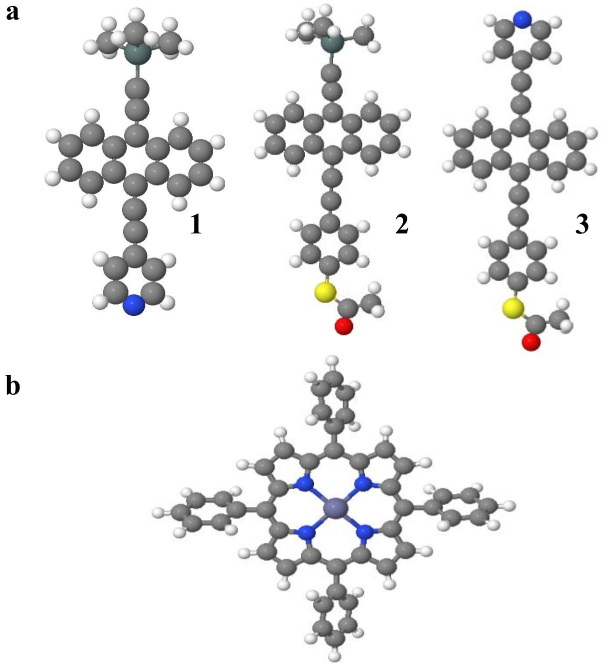


*Zinc-tetraphenylporphyrin (Zn-TPP)*

**Figure SI.14: a**: Asymmetric anthracene-based molecules. **1**: Anthracene with *SnMe$_3$* and *Py* anchors, **2**: Anthracene with *SnMe$_3$* and *SAc* anchors, and **3**: Anthracene with *Py* and *SAc* anchors. **b**: *Zinc tetraphenylporphyrin* (*Zn-TPP*)

### 5.1 Scenarios a:

We employ molecule **1** for this scenario. Figure SI.15 illustrates the components that use to build the flipping junction. It also shows molecule **1** where it consists of spacers and two different anchors groups mainly *SnMe3* and *Py*. Then adding a *Zn-TPP* to form the multicomponent compound. Finally, this structure places between two gold electrodes. To achieve the flipping feature, we first link the *Py* anchor to the *Zn-TPP* from one end and the *SnMe3* to *Au* substrate from the other end and then place this structure between the *Au* electrodes, as shown in the left panel (orientation-1), of Fig. SI.15. It should be noted the *SnMe3* anchor cleaves when it attaches to the gold metal to form an *Au-C* direct contact. Secondly, we flip molecule **1** so that the *SnMe3* anchor is now attached to the *Zn-TPP*, and again place the multicomponent between electrodes as shown the right panel (orientation-1), of Fig. SI.15. We have labelled the two systems as orientation-1 and orientation-2.

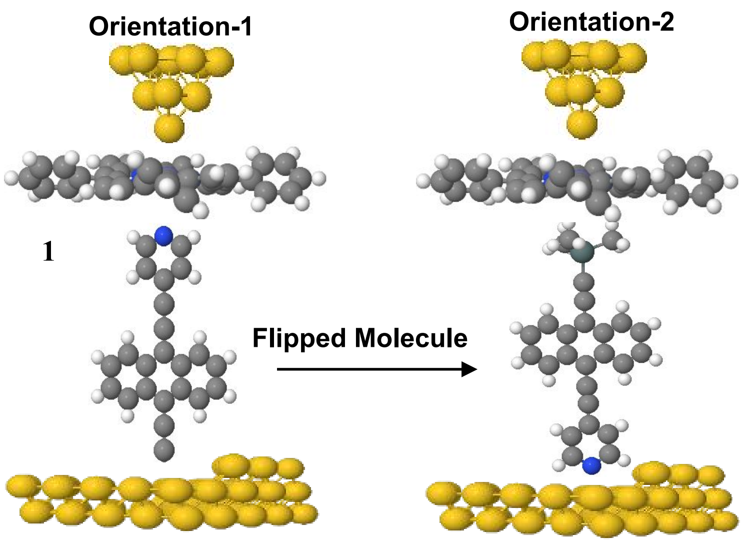


**Figure SI.15:** Schematic illustration of molecular junctions for two orientations of molecule **1**. Orientation-1 and -2 show how molecule **1** flips between the *Zn-TPP* and Au. **Left panel:** Orientation-1 is when the *Py* anchor linked to the *Zn-TPP* from one end and the *TMS* to a *Au* from the other end. **Right panel:** Orientation-2 is the opposite, *SnMe3* anchor linked to the *Zn-TPP* and

We repeat the same simulations that described in section 3, to calculate the transmission coefficient *T(E)*. Top panel of Fig. SI.16 illustrate the transmission coefficient curves for orientation-1 and -2. The two curves demonstrate an opposite behaviour, meaning a HOMO dominated curve for orientation-1 and a LUMO dominated for orientation-2. The Fermi energy locates from the LUMO resonance depends on the orientation of molecule **2** between *Zn-TPP* and *Au*. In other words, how strong the binding to the *Zn-TPP* and *Au* and the type of the anchor. The top panel clearly shows there is a different in the Fermi position when molecule **1** flips from orientation-1 to orientation-2.

Similarly, the Seebeck calculations that described in section 4, apply on the flipping simulations. The lower panel of Fig. SI.16 show the Seebeck coefficient of the two orientations. As the top panel illustrates the two orientations to be HOMO and LUMO dominated then that should reflect in the Seebeck sign, means the curves possess a positive and negative Seebeck.

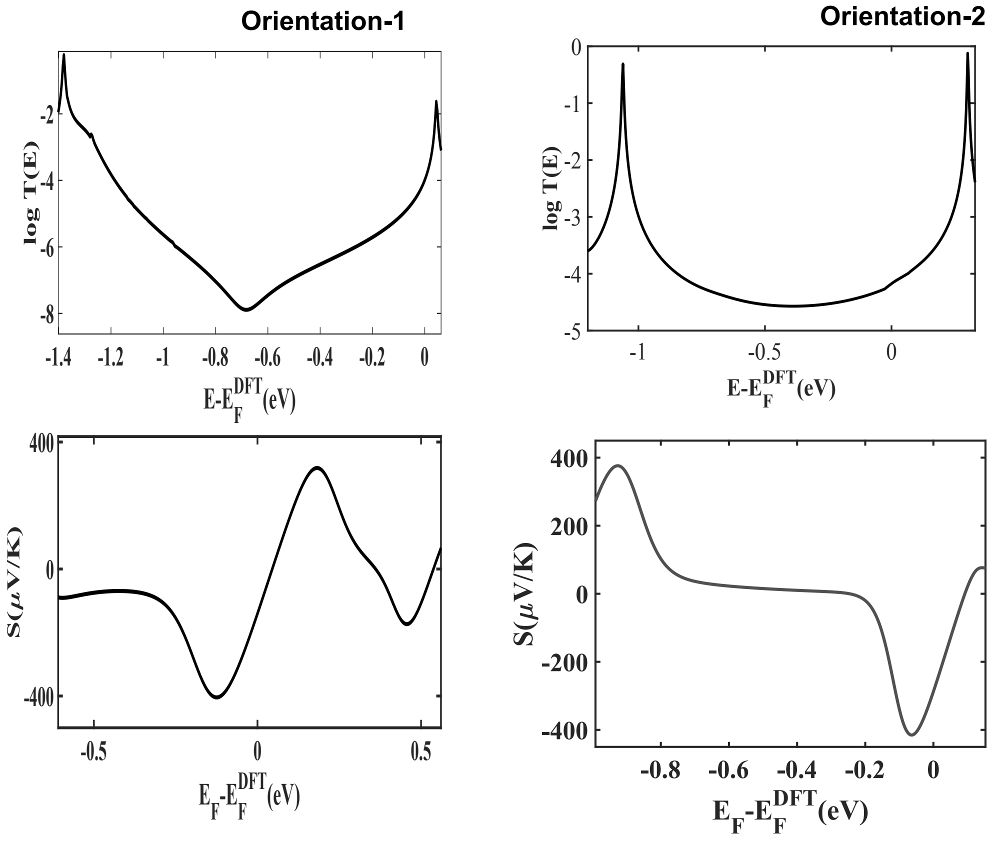


**Figure SI.16: Top panel:** Zero bias transmission coefficients *T(E)* of molecule **1** against electron energy $E$, of orientation-1 and orientation-2 of Fig. SI.15. The flipping feature shifts the Fermi energy $E - E_F^{DFT} = 0$ from 0.07 to 0.13 eV towards a LUMO resonance (left to right respectively). **Lower panel:** Seebeck coefficients $S$ of molecule **1** against electron energy *E,* in two orientations and exhibit a positive and negative Seebeck.

## 5.2 Scenarios b:

This scenario employs molecule **2**, and the same procedure that described in scenario a, repeats however with different anchors. Here, we mainly focus on *SnMe3* and *SAc* although these anchors cleave during the flipping procedure. In orientation-1 the *SnMe3* anchor cleaves from the bottom side to form a *Au-C* direct contact as shown in the left panel of Fig. SI.17. Similarly, the *SAc* anchor cleaves from the bottom side to form a *Au-S* contact as shown in the right panel of Fig. SI.17. Both anchors remain the same when contact the *Zn-TPP*.

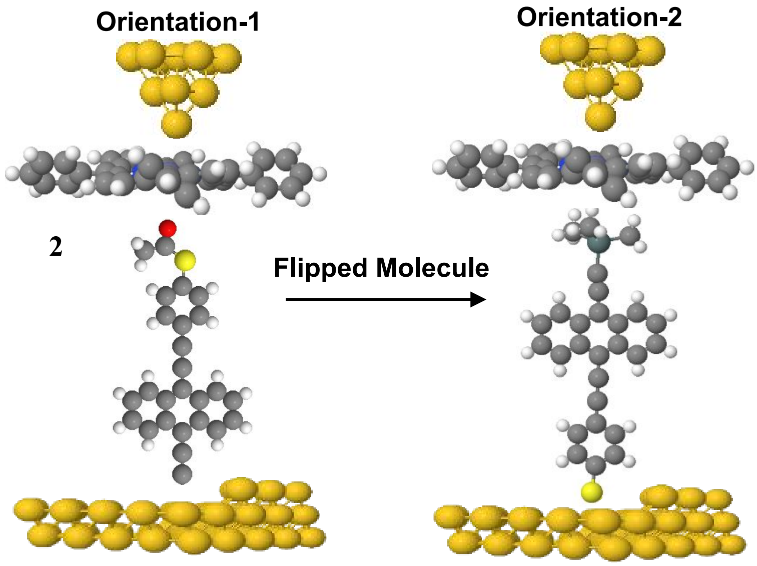


**Figure SI.17:** Schematic illustration of molecular junctions for two orientations of molecule **2**. Orientation-1 and -2 show how molecule **2** flips between the *Zn-TPP* and Au. **Left panel:** Orientation-1 is when the *SAc* anchor linked to the *Zn-TPP* from one end and the *TMS* to a *Au* from the other end. **Right panel:** Orientation-2 is the opposite, *SnMe3* anchor linked to the *Zn-TPP* and *S* anchor to a *Au* contact.

Top panel of Fig. SI.18 illustrate the transmission coefficient curves for orientation-1 and -2 of molecule **2**. The two curves demonstrate a mid-gab curve. the distance between the Fermi energy and the HOMO resonance determines by the orientation of molecule **2** between *Zn-TPP* and *Au*. In other words, how strong the binding to the *Zn-TPP* and *Au* and the type of the anchor. It's clearly shown by the top panel, there is a different in the Fermi position when the molecule flips from orientation-1 to orientation-2.

The lower panel displays the Seebeck calculations of molecule **2**. This panel proves *S* of the two orientations to be a positive. This result is expected as the two orientations are a HOMO dominated curves (see top panel).

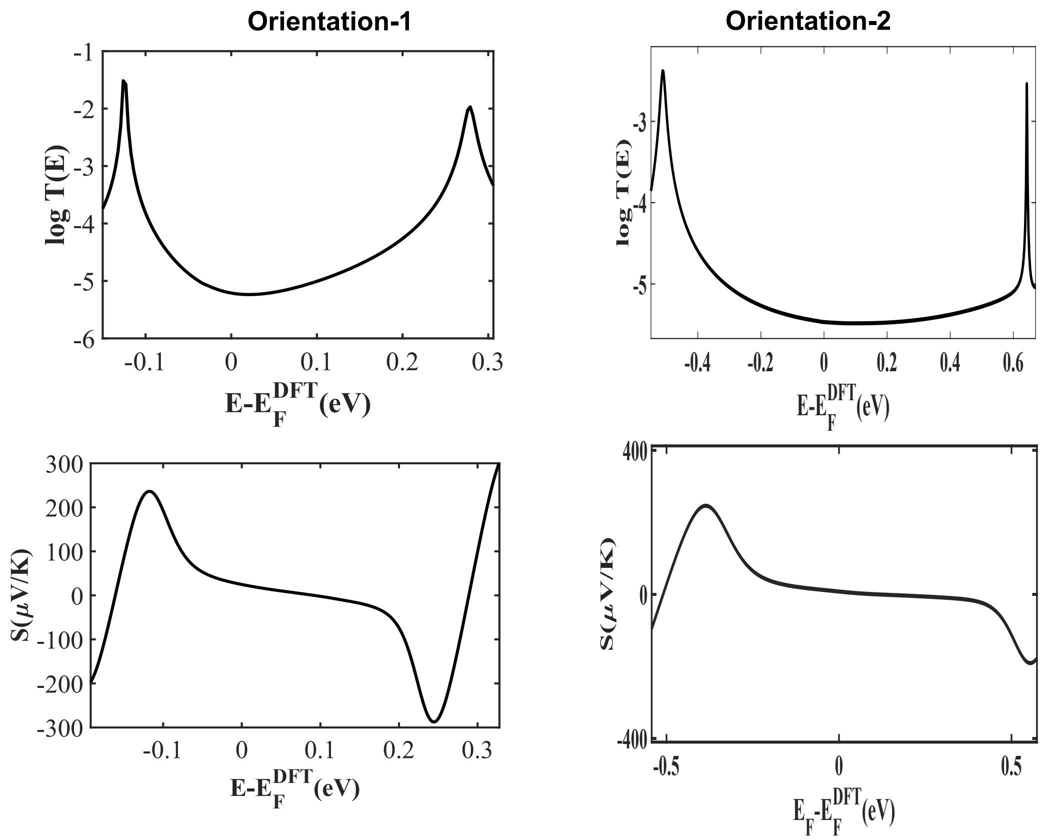


**Figure SI.18: Top panel:** Zero bias transmission coefficients *T(E)* of molecule **2** against electron energy *E*, of orientation-1 and orientation-2 of Fig. SI.15. The flipping feature shifts the Fermi energy $E - E_F^{DFT} = 0$ from 0.01 to 0.15 eV towards a HOMO resonance (left to right respectively). **Lower panel:** Seebeck coefficients *S* of molecule **2** against electron energy *E,* in two orientations and both exhibit a positive Seebeck.

### 5.3 Scenarios c:

Here, we repeat the same procedure that described in scenarios a and b. The difference is the two anchors are *Py* and *SAc,* in this scenario one anchor cleaves during the flipping procedure. Orientation-1 the *SAc* anchor cleaves from the bottom side to form a *Au-S* contact as shown in the left panel of Fig. SI.19. No cleave occurs for orientation-2, where both sides are *Au-Py* and *Zn-TPP -SAc* as shown in the right panel of Fig. SI.19.

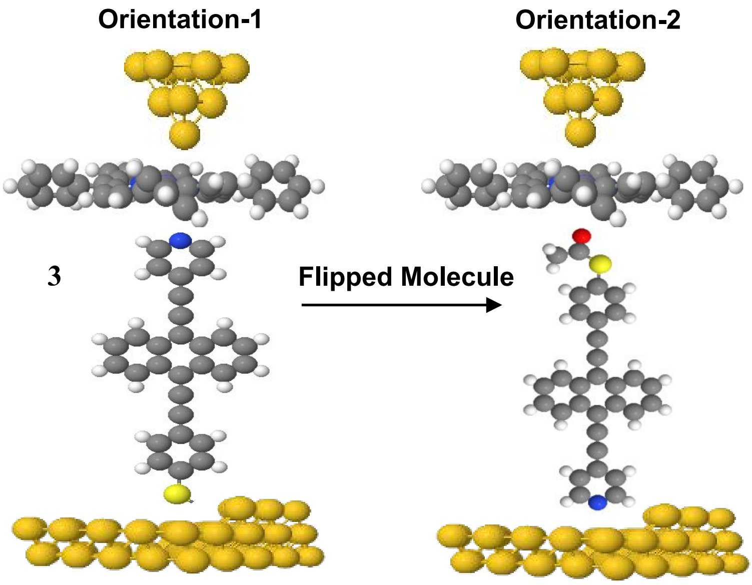


**Figure SI.19:** Schematic illustration of molecular junctions for two orientations of molecule **3**. Orientation-1 and -2 show how molecule **3** flips between the *Zn-TPP* and Au. **Left panel:** Orientation-1 is when the *Py* anchor linked to the *Zn-TPP* from one end and the *S* to a *Au* from the other end. **Right panel:** Orientation-2 is the opposite, *SAc* anchor linked to the *Zn-TPP* and *Py* anchor to a *Au* contact.

Top panel of Fig. SI.20 illustrate the transmission coefficient curves for orientation-1 and -2 of molecule **3**. The two curves demonstrate an opposite behaviour, meaning a HOMO dominated curve for orientation-1 and a LUMO dominated for orientation-2.

Fig. SI.10 investigates molecule **3** transport in gold-gold junction. It illustrates that molecule **3** possesses a mid-gap transport feature. We attribute that to the conflict between a strong HOMO dominated anchor such as thiol and a strong LUMO dominated anchor such as pyridyl. This is clearly distinguished from molecules **1** and **2,** as **1** is a LUMO dominated (see Fig. SI.8), and **2** is a HOMO dominated (see Fig. SI.9), even though both molecules are asymmetric. This finding strongly suggests that one of the anchors overcomes the other, for example, for **1** *Py* > *TMS* and **2** *SH* > *TMS,* therefore, there is either a HOMO or LUMO trend, but not mid-gap likewise **3**.

Now, one would argue that the thiol anchor is stronger than pyridyl in molecule **3** and therefore should obtain a HOMO domination than a mid-gap. To satisfy this concern, there are many studies[10-12] demonstrate the pyridyl anchor is much stronger on a rough Au substrate, thus, we use an ad-atom in our simulations. The second supporting point for this concern is also an experimental evidence (XPS measurements), the percentage of the two orientations as it shall be discussed in the following section.

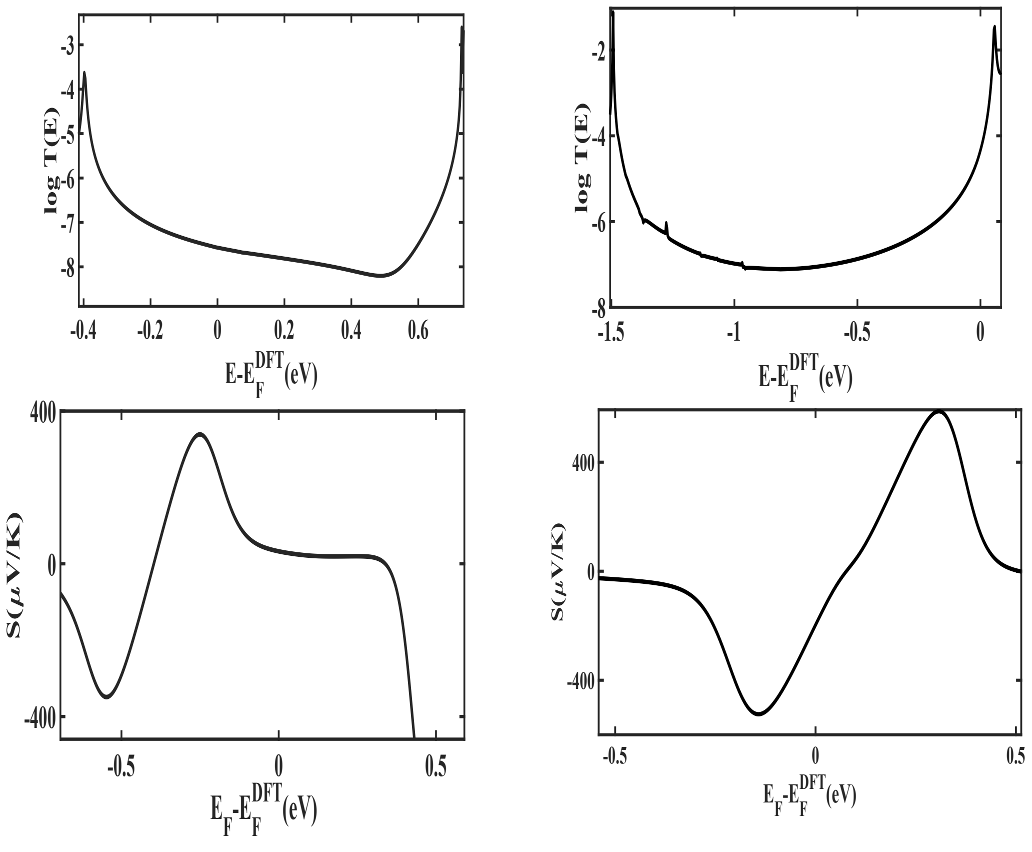


**Figure SI.20: Top panel:** Zero bias transmission coefficients *T(E)* of molecule **2** against electron energy $E$, of orientation-1 and orientation-2 of Fig. SI.18. The flipping feature shifts the Fermi energy $E - E_F^{DFT} = 0$ from 0.01 to 0.15 eV towards a HOMO resonance (left to right respectively). **Lower panel:** Seebeck coefficients $S$ of molecule **2** against electron energy *E,* in two orientations and both exhibit a positive and negative Seebeck.

## 6. References


(1) Soler, J. M.; Artacho, E.; Gale, J. D.; García, A.; Junquera, J.; Ordejón, P.; Sánchez-Portal, D. J. J. o. P. C. M., The SIESTA method for ab initio order-N materials simulation. *J. Phys.: Condens. Matter* **2002,** *14* (11), 2745.

(2) Emilio, A.; Anglada, E.; Diéguez, O.; Gale, J. D.; García, A.; Junquera, J.; Martin, R. M.; Ordejón, P.; Pruneda, J. M.; Sánchez-Portal, D.; Soler, J. M., The SIESTA method; developments and applicability. *J. Phys.: Condens. Matter* **2008,** *20* (6), 064208.

(3) Kobko, N.; Dannenberg, J., Effect of basis set superposition error (BSSE) upon ab initio calculations of organic transition states. *The Journal of Physical Chemistry A* **2001,** *105* (10), 1944-1950.

(4) Sherrill, C. D., Counterpoise correction and basis set superposition error. *School of Chemistry and Biochemistry, Georgia Institute of Technology* **2010**.

(5) Chen, W.; Widawsky, J. R.; Vázquez, H.; Schneebeli, S. T.; Hybertsen, M. S.; Breslow, R.; Venkataraman, L., Highly conducting π-conjugated molecular junctions covalently bonded to gold electrodes. *Journal of the American Chemical Society* **2011,** *133* (43), 17160-17163.
(6) Ismael, A. K.; Lambert, C. J., Single-molecule conductance oscillations in alkane rings. *J. Mater. Chem. C,* **2019,** *7*, 6578-6581.
(7) Wang, C.; Batsanov, A. S.; Bryce, M. R.; Martin, S.; Nichols, R. J.; Higgins, S. J.; Garcia-Suarez, V. M.; Lambert, C. J., Oligoyne single molecule wires. *Journal of the American Chemical Society* **2009,** *131* (43), 15647-15654.
(8) Obersteiner, V.; Egger, D. A.; Zojer, E., Impact of anchoring groups on ballistic transport: single molecule vs monolayer junctions. *The Journal of Physical Chemistry C* **2015,** *119* (36), 21198-21208.
(9) Bennett, T. L.; Alshammari, M.; Au-Yong, S.; Almutlg, A.; Wang, X.; Wilkinson, L. A.; Albrecht, T.; Jarvis, S. P.; Cohen, L. F.; Ismael, A., Multi-component self-assembled molecular-electronic films: towards new high-performance thermoelectric systems. *Chem. Sci.* **2022,** *13* (18), 5176-5185.
(10) Hou, S. M.; Zhang, J. X.; Li, R.; Ning, J.; Han, R. S.; Shen, Z. Y.; Zhao, X. Y.; Xue, Z. Q.; Wu, Q., First-principles calculation of the conductance of a single 4,4 bipyridine molecule. *Nanotechnology* **2005,** *16* (2), 239-244.
(11) Quek, S. Y.; Kamenetska, M.; Steigerwald, M. L.; Choi, H. J.; Louie, S. G.; Hybertsen, M. S.; Neaton, J.; Venkataraman, L., Mechanically controlled binary conductance switching of a single-molecule junction. *Nat Nanotechnol* **2009,** *4* (4), 230-234.
(12) Stadler, R.; Thygesen, K. S.; Jacobsen, K. W., Forces and conductances in a single-molecule bipyridine junction. *Physical Review B* **2005,** *72* (24), 241401.
